# Identification and enumeration of circulating tumor cells in the cerebrospinal fluid of breast cancer patients with central nervous system metastases

**DOI:** 10.18632/oncotarget.336

**Published:** 2011-10-08

**Authors:** Akshal S. Patel, Joshua E. Allen, David T. Dicker, Kristi L. Peters, Jonas M. Sheehan, Michael J. Glantz, Wafik S. El-Deiry

**Affiliations:** ^1^Laboratory of Translational Oncology and Experimental Cancer Therapeutics, Department of Medicine (Hematology/Oncology), Penn State Hershey Cancer Institute, Penn State College of Medicine, Hershey, PA, USA; ^2^Department of Neurological Surgery, Penn State Hershey Medical Center, Hershey, PA, USA; ^3^Biochemistry and Molecular Biophysics Graduate Group, University of Pennsylvania School of Medicine, Philadelphia, PA, USA

**Keywords:** circulating tumor cells, carcinomatous meningitis, breast cancer, brain metastases, intrathecal chemotherapy, cancer monitoring

## Abstract

The number of circulating tumor cells (CTCs) in the peripheral blood of metastatic breast cancer patients is now an established prognostic marker. While the central nervous system (CNS) is a common site of metastasis in breast cancer, the standard marker for disease progression in this setting is cerebrospinal fluid (CSF) cytology. However, the significance of CSF cytology is unclear, requires large sample size, is insensitive and subjective, and sometimes yields equivocal results. Here, we report the detection of breast cancer cells in CSF using molecular markers by adapting the CellSearch system (Veridex). We used this platform to isolate and enumerate breast cancer cells in CSF of breast cancer patients with central nervous system metastases. The number of CSF tumor cells correlated with tumor response to chemotherapy and were dynamically associated with disease burden. This CSF tumor cell detection method provides a semi-automated molecular analysis that vastly improves the sensitivity, reliability, objectivity, and accuracy of detecting CSF tumor cells compared to CSF cytology. CSF tumor cells may serve as a marker of disease progression and early-stage brain metastasis in breast cancer and potentiate further molecular analysis to elucidate the biology and significance of tumor cells in the CSF.

## INTRODUCTION

In the modern era of oncology, cancer-related mortality is associated with metastatic spread rather than the voraciousness of the primary tumor [4]. Tumor seeding in secondary tissues therefore presents a major challenge in cancer treatment [5]. Therapeutic strategies are often focused on tumor containment in lieu of concerns for tumor cell dissemination into surrounding structures. The enumeration of circulating tumor cells (CTCs) can monitor the metastatic potential of some solid tumors, relate these cells to patient survival, and provide a surrogate marker of treatment response [6, 7]. Giving rise to the “liquid biopsy”, peripheral blood can be analyzed for the presence of CTCs using a range of techniques that are in various stages of development [8]. The CellSearch system is a CTC detection method that utilizes several molecular parameters to isolate CTCs: immunomagnetic enrichment for epithelial cell adhesion molecule (EpCAM), nuclear staining with 4′, 6-diamidino-2-phenylindole (DAPI), and immunofluorescence detection of cytokeratin and CD45 [9]. Due to its demonstrated reliability and prognostic value, the CellSearch system is the only CTC detection platform approved by the US Food and Drug Administration (FDA) for the enumeration of CTCs in metastatic colorectal, prostate, and breast cancers.

The cerebrospinal fluid (CSF) is an important, unique, and poorly understood compartment of the central nervous system (CNS). CSF often acts as a biologic sump for neurons and glia and is continuously produced and recycled much like blood or lymph, though never filtered. Cells from solid tumors can infiltrate the CSF by several mechanisms including blood-brain barrier penetration by circulating cells, directly through tumor extension along Vichow-Robin spaces, or through patterned secondary structures of Scherer [10, 11]. Tumor cells within the CSF represent a special subpopulation of malignant cells that have proven their metastatic potential in peripheral blood and may be a source of a number of devastating neurologic sequelae. Current methods for examining the CSF involve pathological identification of abnormal cells by Wright-Giemsa stain. With this method there is no quantification or characterization of these cells and clinicians must make judgments on the binary presence or absence of malignant cells as determined by cytology [12]. This is the gold standard but lacks molecular analysis and sensitivity, often requiring repeat testing or high volume analysis [13].

The prognosis and therapeutic stratification of patients with CNS metastases is currently based on the integration of histopathologic data, the appearance and severity of neurologic symptoms, and magnetic resonance imaging (MRI) of the neuroaxis [14]. To overcome limitations of currently available clinical parameters, we developed a reliable detection method to enumerate CSF tumor cells (CSFTCs) using molecular tumor cell markers by adapting the CellSearch system. This report describes this detection method and demonstrates the feasibility and significance of CTC detection in a pilot study of metastatic breast cancer patients with CNS metastases.

## RESULTS

### Selective detection of breast cancer cells

**Figure 1:**
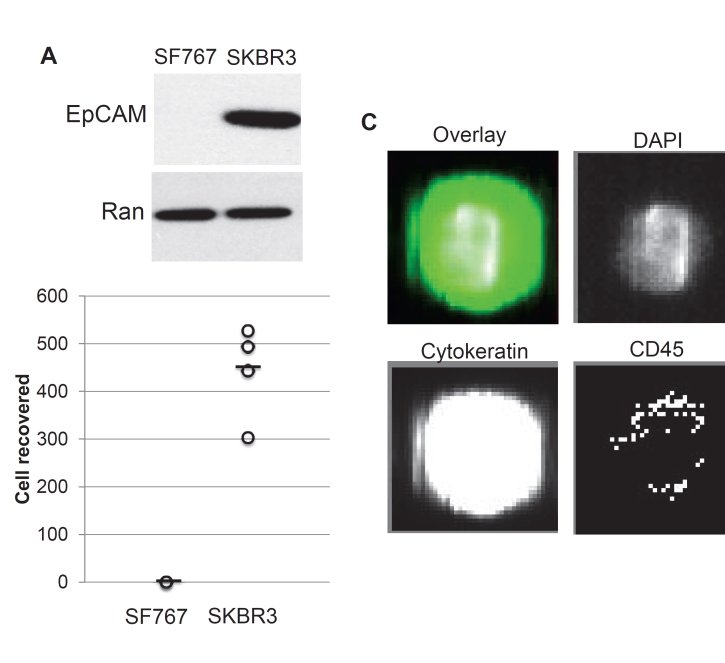
Detection by the CellSearch system of breast cancer cells but not glioblastoma cells present in human blood (A) Western blot analysis of EpCAM expression in SF767 human glioblastoma cells and SKBR3 human breast cancer cells. Ran shown as a loading control. (B) Number of CTCs enumerated by CellSearch criteria in normal human blood spiked with 1,000 SF767 and SKBR3 cells from cell culture. (C) Exemplary image of an SKBR3 cell isolated by the CellSearch system.

One of the diagnostic criteria for CTCs as defined by the CellSearch system is to be EpCAM^+^. We found that human glioblastoma cells do not express EpCAM contrary to breast cancer cells as expected (Figure 1A). Accordingly, spiking these cultured cells into normal human blood revealed that the CellSearch system detects breast cancer cells but not glioblastoma cells (Figure 1B-C). This suggests that the CellSearch system could be used to detect cancer cells in the CSF that are not of glial origin and therefore those with metastatic potential to the CNS.

### Adapting the CellSearch system to detect CSFTCs

**Figure 2:**
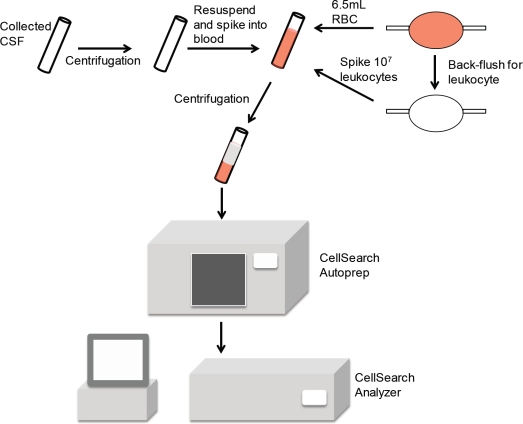
Schematic demonstrating use of the CellSearch system to detect breast cancer cerebrospinal fluid tumor cells

We aimed to develop a method of using the CellSearch system to detect tumor cells in CSF. The CellSearch uses 7.5 mL of peripheral blood for its standard detection method and relies on several checkpoints and caveats throughout its processing. One such caveat is that the tumor cells will be in the buffy coat following centrifugation of the sample. Due to this, CSF cannot be directly analyzed in the machine in lieu of blood. We circumvented this by spiking the cellular contents of the CSF sample into normal human blood for detection. This was accomplished by centrifugation of the CSF and resuspension in a small volume of phosphate buffered saline (Figure 2). We regularly obtained normal blood for these assays by procuring leukocyte filters from blood drives and reconstituting normal blood with a physiological number of leukocytes. The CSF suspension was then spiked into the reconstituted blood and subjected to the standard CellSearch protocol. It is noteworthy that leukocytes are particularly important for this assay as the CellSearch system relies on CTCs and contaminating leukocytes when determining the focal plane for immunofluorescence analysis. Therefore if a sample with no CTCs is analyzed, the CellSearch system will abort the analysis if there are no remaining cells present for locating the focal plane.

### Accuracy and reproducibility of detecting CSFTCs

**Figure 3:**
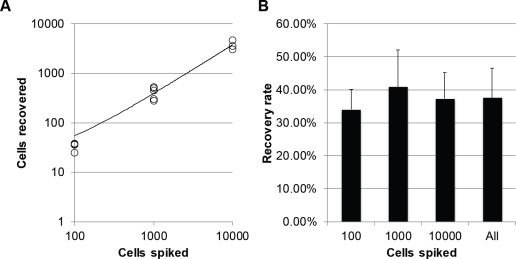
Recovery rate of breast cancer cells present in cerebrospinal fluid (A) Enumeration and (B) recovery rate of cultured SKBR3 cells spiked into normal blood (n>3).

To characterize the recovery rate and linearity of recovery, we spiked cultured breast cancer cells at varying cell numbers and determined the recovery rate at these various cell counts (Figure 3). We found that the recovery rate was ~38%, which is similar to that found in other recovery experiments where cultured human tumor cells are spiked directly into blood (data not shown). We also found this recovery rate to be reproducible and linear across the range of cell numbers tested (R^2^ = .96).

### Detection of CSFTCs in breast cancer patients with CNS metastases and association with disease burden

**Figure 4:**
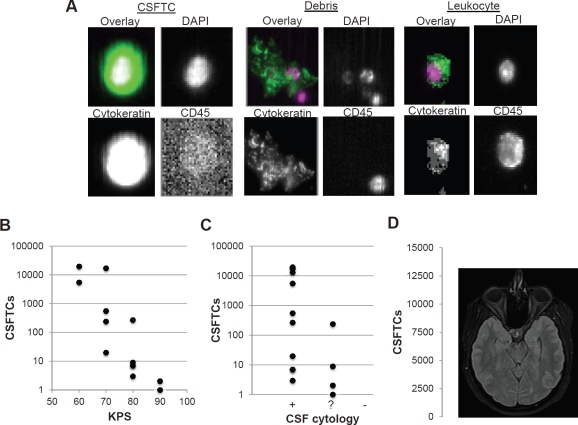
Detection of CSFTCs in breast cancer patients with CNS metastasis and correlation with Karnofsky performance status (A) Exemplary images of species detected in CSF samples. (B) Correlation of Karnofsky performance status with CSF cytology versus CSFTC number in subjects 1,2, 3, and 5 throughout treatment. (C) Correlation of CSFTCs with CSF results that report positive (+), equivocal (?), or negative (-). (D) MRI of subject 5 on day of CSF withdrawal.

Five patients with metastatic breast cancer involving the CNS were enrolled into a pilot study (Table 1). Detecting CSFTCs yielded a number of morphologically diverse species that included leukocytes, tumor cells, and significant amount of debris (Figure 4A). Note that debris is easily separable from tumor cells due to the immunostaining for a tumor cell marker and is an important advantage because tumor cells may be more difficult to distinguish by CSF cytology. Analyzing data at baseline and following treatment, there was a general inverse correlation between Karnofsky performance status (KPS) of and CSFTC number (*r* = -.66) (Figure 4B). There was a general trend toward a higher CSFTC count with positive CSF cytology (r = -.37) (Figure 4B), though CSF cytology clearly does not reflect disease burden (Figure 4C). Subject 5 had >12,000 CSFTCs and on the same day of CSF collection developed status epilepticus and deceased. Interestingly this patient showed no gross abnormalities by MRI that suggested tumor burden and this underscores the importance of CSFTCs as a disease marker (Figure 4D). While the correlation between CSFTC number and Karnofsky performance shows significance in the pilot study, a large cohort of patients will likely reveal a stronger significance than can be demonstrated with this pilot study.

**Table 1:** Patient characteristics

Subject	Age	Her2	Progesterone receptor	Estrogen receptor	Baseline KPS	Chemotherapy
1	54	+	-	-	80	Intrathecal trastuzumab, liposomal cytarabine, methotrexate
2	52	-	-	+	70	Intrathecal liposal cytarabine, methotrexate, thiotepa, topotecan
3	28	-	-	-	60	Intrathecal liposal cytarabine, methotrexate, thiotepa, topotecan
4	34	-	-	-	90	Intrathecal thiotepa, methotrexate, topotecan
5	35	-	-	-	60	Intrathecal thiotepa and topotecan

### CSFTC count dynamically changes with treatment

**Figure 5:**
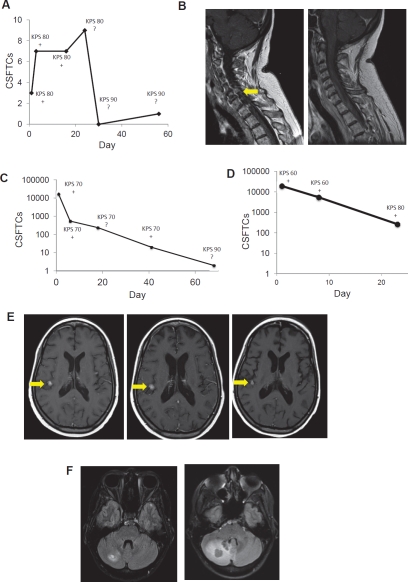
CSFTC counts dynamically change with chemotherapy (A) CSFTC count over time and (B) MRI of subject 1 at baseline and days post-treatment initiation. CSFTC count over time in (C) subject 2 and (D) subject 3. (E) MRI of subject 3 at baseline (left panel), 42 (middle panel), and 68 days (right panel) post-treatment initiation. (F) MRI of subject 4 at baseline (left panel) and 57 days post-treatment initiation (right panel).

Response to chemotherapy was evident in three patients that were followed over time in this pilot study (Figure 5). Subjects 1, 2, and 3 exhibited a significant decline in the number of CSFTCs following initiation of intrathecal therapy consisting of a combination of topotecan, liposomal cytarabine, thiotepa, methotrexate and/or trastuzumab (Table 1). It should be noted that at several time-points CSF cytology conducted by a blinded pathologist gave an equivocal result and that improvements in KPS tended to follow a decline in CSFTCs. This data clearly highlights the inability of CSF cytology to reflect dynamic changes in disease burden.

In addition to its general correlation with KPS, changes in CSFTC number tended to coincide with clinical deficits. Prior to treatment initiation, subject 2 had >10^4^ CSFTCs and exhibited facial weakness and vertical diplopia. These symptoms were resolved approximately two months after treatment and was accompanied by a near complete disappearance of CSFTCs. Subject 3 also harbored >10^4^ CSFTCs and suffered from severe vomiting, lethargy, and headaches. Following treatment this patient had a CSFTC count that declined significantly to 267 CSFTCs but still suffered from headaches, though other symptoms resolved. The clinical outcome of leptomeningeal or parenchymal CNS metastases varies greatly. Patients with an excess of 10,000 CSFTCs had cranial nerve deficits or mental status decline. Two patients showed an improvement in KPS after chemotherapy initiation and a concomitant decline in CSFTCs. Our findings from this small pilot study suggest a possible predictive role for CSFTCs and their quantification to serve as a reliable marker that reflects disease burden dynamically unlike CSF cytology and provides information regarding the magnitude of disease burden.

Gadolinium-enhanced MRI allows for the sensitive visualization of macroscopic changes with the brain, spine or leptomeninges. We found an inverse correlation with CSFTC count and the presence or enhancement pattern seen on such imaging. It should be noted that though neuroimaging is an indicator of CNS disease, it relies on the breakdown of the blood-brain barrier rather than a direct sign of response. Multi-modality imaging is an ever-advancing technology but is not sufficient to make judgments about cranial and spinal metastases. CSFTCs represent a novel marker of CNS disease that detects cancer at the single cell level and provides critical information to clinicians that attempt to combine cytology, imaging and neurologic examination as part and parcel of a treatment algorithm. While this study serves as a proof-of-principle and shows promising significance, a larger cohort of patients with follow up studies will be required to concretely establish the prognostic significance of CSFTC enumeration and the ability of therapeutic agents to eliminate these cells.

## DISCUSSION

The biology of metastases and their relationship to the microenvironment of the CNS remains unclear [15, 16]. The nature and purpose of malignant cells from extraneural sites within the CSF compartment also remains unexplored [17]. Our investigation suggests that the CSF is a viable source for detecting metastatic tumor cells in cancer patients with CNS involvement. Micrometastases to the cerebrospinal compartment and transmigration through the blood-brain barrier are poorly understood events[17-20]. Current anti-cancer therapies are relatively successful for local control of breast cancer at early stages. However, late-stage and recurrent breast cancer often metastasizes to the brain [21].

Accurate, early diagnosis and appropriate treatment decisions are likely to yield a better patient outcome and underscores the importance of accurately monitoring common secondary sites by a highly sensitive detection method. The ability to detect single cells at a metastatic site such as the brain may allow for therapeutic intervention that could prevent or destroy metastatic disease an early stage [22]. Delays in diagnosis can be disastrous from a neurologic standpoint and treatment decisions must weigh the risks of aggressive treatment with the extent of disease. As potentiated by the detection of CSFTCs, the knowledge that viable cancer remains in the CSF of patients is highly useful in making treatment decisions.

A number of techniques have been developed for the isolation of CTCs in peripheral blood since the first attempts in the late 1800s [23]. Investigational labs now use several different techniques for CTC detection including reverse transcriptase polymerase chain reaction, immunocytochemistry, flow cytometry, microchips, and size-based filtration methods [24-26]. The CellSearch system presents a platform that reliably captures CTCs in this setting and provides a semi-automated platform for enumerating CTCs based on multiple markers to yield high accuracy, recovery, and reproducibility. Several studies have demonstrated the clinical significance of CTC number as a prognostic marker when enumerated by the CellSearch system in metastatic tumors of breast, colon and prostate [1, 27, 28]. The isolation and enumeration of CSFTCs may provide valuable information for patients with CNS metastasis and potentiate studies on the biology of these cells and how they differ from the primary and metastatic tumor as well as the CTCs found in peripheral blood.

The prognostic relevance of CSFTCs is correlated here with the clinical course of the patient and the CSFTC count. The biological significance of finding up to several thousand CSFTCs in a patient sample remains unclear though it is suggestive of and correlates with high disease burden. CNS metastases are often strategically located in close proximity to ventricular surfaces or CSF cisterns. However, natural circulation of CSF can be impaired, which may cause “loculations” of malignant CSF, thus lowering the diagnostic yield of lumbar puncture [29].

Our lab is currently pursuing post-isolation characterization of tumor cells isolated from peripheral blood as well as CSF [30]. There is an expanding though controversial body of evidence that cancer stem cells play a large role in the propagation of solid tumors, including critical mechanisms of metastasis [31-33]. The reliable, sensitive, and accurate isolation of CSFTCs enables such studies in conjunction with is potential as a novel marker that has clear advantages over CSF cytology.

## METHODS

### Cell culture experiments

Human SKBR3 breast cancer cells were obtained from ATCC and cultured in McCoy's 5A medium supplemented with 10% heat-inactivated fetal bovine serum and 1% penicillin and streptomycin. Human SF767 glioma cells were a kind gift from Akiva Mintz (Wake Forest University) and were cultured in RPMI under the same conditions. For Western blot analysis, cells were harvested by cell scraping, centrifuged, and lysed on ice for 2 hours. Lysates were harvested and the protein concentration was determined using the BioRad protein assay. Samples were electrophoresed on 4-12% Bis-Tris gels, transferred to PVDF, and blocked in 10% non-fat milk in TBST. Membranes were incubated with EpCAM (Cell Signaling) at 1:500 or Ran at 1:10,000 (BD biosciences) antibodies overnight at 4°C. Membranes were rinsed in TBST, incubated with an appropriate HRP-conjugated secondary antibody, and visualized using ECL-Plus and X-Ray film.

### Cell spiking experiments

Tumor cells were harvested from log-phase growth by trypsinization and enumerated using a Cellometer (Nexcelom Biosciences) in triplicate. The appropriate volume of cell suspension was then added to a tube of CSF previously cleared of tumor cell contaminants by centrifugation. This CSF was then subjected to the standard CSFTC detection procedure described below.

### Patients

The main inclusion criteria were newly discovered breast cancer with metastatic disease involving the CNS as confirmed with radiologic or cytologic findings and the commencement of intrathecal chemotherapy. All subjects provided informed consent for testing of their CSF as approved by the Institutional Review Board at the Penn State Hershey Medical Center. All subjects were diagnosed with primary breast cancer prior to enrollment into this study and had undergone neurosurgical intervention in terms of placement of a ventricular access device. All patients had involvement of the CNS, with combinations of parenchymal or leptomeningeal metastases. The ventricular access device provided a means to obtain CSF for testing. Prior to the onset of treatment, patients underwent thorough neurologic examination to identify any deficits and were stratified based on KPS. CSF was obtained every 2 to 3 weeks for each patient and often coincided with intrathecal chemotherapy deposition. The neuro-oncologist providing treatment and evaluating the patients was blinded to the CSFTC analysis.

### Detection of CSFTCs

9mL of CSF was centrifuged at 500g for 10 minutes, the supernatant was removed, and the pellet was resuspended in 1mL of PBS. The suspension was then spiked into 6.5mL of reconstituted normal human blood. This blood was obtained from leukocyte filters that were generated from blood drives as approved by the Institutional Review Board at Penn State Hershey Medical Center. 6.5mL of filtered blood was removed from the leukocyte trap, placed into a CellSave (Veridex) tube, and inverted 8 times. The leukocyte filter was rinsed with PBS and back-flushed to obtain leukocytes that were enumerated and spiked at 10^7^ cells per 6.5mL sample of blood. Samples were analyzed within 3 days using the standard CellSearch protocol and the CTC Epithelial Cell Kit (Veridex). In brief, the CellSearch system qualifies a cell as a CTC if it has an evident nucleus by DAPI staining and is EpCAM^+^, cytokeratin^+^, and CD45^-^. Analysis and enumeration of CTCs was conducted by a blinded, certified assay operator.

### Statistics

Bivariate correlation calculated by Pearson's correlation.
